# High Schizotypal Individuals Are More Creative? The Mediation Roles of Overinclusive Thinking and Cognitive Inhibition

**DOI:** 10.3389/fpsyg.2018.01766

**Published:** 2018-09-21

**Authors:** Lixia Wang, Haiying Long, Jonathan A. Plucker, Qing Wang, Xiaobo Xu, Weiguo Pang

**Affiliations:** ^1^Shanghai Teacher Training Center, Shanghai, China; ^2^Institute of Developmental and Educational Psychology, School of Psychology and Cognitive Science, East China Normal University, Shanghai, China; ^3^Leadership and Professional Studies, Florida International University, Miami, FL, United States; ^4^The Center for Talented Youth and School of Education, Johns Hopkins University, Baltimore, MD, United States

**Keywords:** creativity, cognitive inhibition, intelligence, overinclusive thinking, schizotypy, shifting, working memory

## Abstract

Although a theoretical link between positive schizotypy and heightened creativity has been established in the literature, little empirical research has been conducted to examine the underlying cognitive processes that contribute to this association. In addition, previous studies found a negative relationship between positive schizotypy and cognitive inhibition; however, they often used the paradigm of latent inhibition. This study used the paradigm of prepotent response inhibition indicated by Stroop interference effect and examined the mediation effects of overinclusive thinking (OT) and cognitive inhibition on the creativity of schizotypal individuals. Two groups of low and high schizotypal individuals (*N* = 78) participated in the study. Each participant completed one OT task, one color-word Stroop task, three other executive functioning (EF) control tasks, and two creativity tasks. The results indicated that the high schizotypal group outperformed the low schizotypal group in the creativity tasks. They also exhibited higher OT as indicated by faster reaction time and higher cognitive inhibition as indicated by lower Stroop interference effect. Further, participant’s levels of OT and cognitive inhibition partially mediated the relationship between schizotypy and creativity. The results were discussed under the context of schizotypy and creativity research and implications for rehabitation were further provided.

## Introduction

The relationship between psychopathology and creativity has been a topic of interest for researchers over the last five decades (Karlsson, 1970; Hasenfus and Magaro, 1976). Researchers have consistently suggested that, overall, there is a statistically significant correlation between the two variables; however, the direction and strength of the relationship depends on many factors, such as the specific symptoms of psychopathology, measures of creativity, and types of creativity (Jones et al., 2011; Fink et al., 2014a; Paek et al., 2016; Taylor, 2017). For instance, some researchers have indicated a negative relationship between the two variables, that is, less severe symptoms of psychopathology are significantly correlated with higher level of creativity (Claridge and Blakey, 2009; Barrantes-Vidal, 2014; Fink et al., 2014a). Although other researchers agree with this notion, they further noted that the two variables has an inverted-U relationship, that is, the mild expressions of psychopathology may facilitate creativity but its full symptoms may hinder it (Acar et al., 2018). Some researchers have demonstrated that people who are prone to psychosis characterized by delusion, hallucination, and negative symptoms show more creativity (Heckers et al., 2013; Fink et al., 2014a). In a recent meta-analysis of 32 studies, researchers have found that the overall mean effect size of the association between creativity and psychoticism is small but the large effect size only shows when psychoticism is measured by the Eysenck Personality Questionnaire and uniqueness is an indicator of creativity (Acar and Runco, 2012). In addition, researchers showed that the negative relationship between schizophrenia and creativity becomes stronger among patients of chronic schizophrenia (Jaracz et al., 2012; Acar et al., 2018) and when creativity is measured by semantic or verbal-letter fluency tasks (Acar et al., 2018). Further, previous studies have revealed that those having schizophrenia, bipolar disorder, or unipolar depression and their relatives were overrepresented in creative occupations and that those with schizophrenia show more artistic and writing creativity (Kyaga et al., 2011, 2013; Rybakowski and Klonowska, 2011).

Similar to the notion that the relationship between psychopathology and creativity depends on the symptoms and severity of psychopathology, researchers have indicated that, rather than schizophrenia, it is schizotypy^1^, a personality trait similar to schizophrenia symptoms but at a diminished level (Debbane and Mohr, 2015), that explains general creativity^2^ and creative performance (Kaufman and Paul, 2014; Fisher, 2015; Wang et al., 2017). While schizophrenia is a psychiatric disorder, schizotypy is a psychological construct that is characterized by the personality traits, such as magical ideation (the propensity to have non-conventional beliefs and accept causality not culturally valid), perceptual aberration (the distorted perception of body and objects), anhedonia, social withdrawal, eccentric behavior, and odd speech (Schuldberg et al., 1988; Cox and Leon, 1999; Nelson et al., 2013). Among these characteristics of schizotypy, magical ideation and perceptual aberration are viewed as positive schizotypy, whereas anhedonia and social withdrawal are viewed as negative schizotypy (Chen et al., 1997; Cox and Leon, 1999; Grimshaw et al., 2010; Rominger et al., 2013). This categorization is consistent with the previous findings that proneness to approach-based psychopathologies (e.g., positive schizotypy and risk of bipolar disorder) are positively related with creativity, whereas proneness to avoidance-based psychopathologies (e.g., anxiety, negative schizotypy, and depressive mood) are negatively related with creativity (Baas et al., 2016).

A substantial amount of studies has shown that individuals with higher levels of schizotypal personality traits attain higher creative achievement and creative performance in assessment tasks. For instance, in one study, visual artists were reported to score significantly higher than the non-artists in all the measures of schizotypy and divergent thinking tasks (Burch et al., 2006). In another study, positive correlations were found in the following relationships: self-rated creativity and unusual experience aspect of schizotypy measured by the Oxford-Liverpool Inventory of Feelings and Experiences (O-LIFE: Mason et al., 1995); creative personality measured by Creative Personality Scale (Gough, 1979), creative achievement measured by Biographical Inventory of Creative Behaviors (BICB, Batey, 2007) and impulsive non-conformity of schizotypy; the total creativity aggregated by the three measures (self-rated, CPS, and BICB) and unusual experiences and impulsive non-conformity (Beaty and Furnham, 2008). A more recent study also found that high schizotypal individuals showed significant advantages over low schizotypal individuals in both verbal (Alternative Uses Test) and figural (Figure Completion and Extraterrestrial Drawing) DT tasks (Wang et al., 2017).

Although the relationship between schizotypal traits and creative performance seems to be well-established, little research has directly explored the cognitive underpinnings of the relationship (Crabtree and Green, 2016). One study examined the common factors that predispose an individual to both creativity and psychosis and indicated that overinclusive thinking (OT) and cognitive inhibition may function as the cognitive link between schizotypy and creativity (Acar and Sen, 2013). This is in line with the underlying cognitive process of the dual process model that has been discussed in the field of creativity. That is, engaging in creative tasks may involve both automatic or associative process and effortful or controlled process (Schmajuk et al., 2009; Beaty et al., 2014, 2016; Edl et al., 2014; Forthmann et al., 2016). However, to date, very few studies have examined the effects, particularly mediation effects, of OT and cognitive inhibition, on the relationship of schizotypy and creativity. The current study attempts to fill this gap in the literature.

## Literature Review

### Overinclusive Thinking, Schizotypy, and Creativity

Overinclusive thinking is usually conceptualized as the inability to preserve conceptual boundaries and identified as a cognitive characteristic of individuals with schizotypy who show an over-responsiveness to associative or irrelevant aspects of words and extraneous stimuli (Payne and Friedlander, 1962). People with OT tend to have a broader conceptual boundary. For example, when answering the questions in categorization tasks, such as “Are feet vehicles?”, people with OT tend to think of feet as vehicles based on the fact that feet transport people and items from one place to another just like vehicles. However, people without OT would not place feet in the vehicle category because they believe that wheels are the necessary features of vehicles (Chiu, 2015). Prior research has also recognized loose associative processing, or allusive thinking, as a feature of the cognitive processes of individuals with schizophrenia spectrum (Meehl, 1990). An empirical study that evaluated predisposing factors related to cognitive control further revealed that common components of positive schizotypy may underlie the disposition to perceive meaningful coincidences and to engage in loose associative processing (Rominger et al., 2011). Another study focusing on the neuropsychological functioning of individuals with schizotypal traits also indicated that, when compared to the healthy control group, high schizotypal individuals demonstrated decreased ability in conceptualization (Kim et al., 2011).

Past research on the nature of creativity has further suggested that the schizotypal trait of OT may play a fundamental role in creative cognition (Eysenck, 1993; Mohr et al., 2001). For instance, the dual process theory of creative cognition indicates that both associative and executive processes are involved to produce novel ideas (Mednick, 1962; Benedek et al., 2012b; Beaty et al., 2014; Forthmann et al., 2016). A study relating creativity to personality proposed that OT may play a leading role in creativity during mental searching processes by providing individuals with more ideas and increasing the possibility of producing creative ideas (Eysenck, 1993; Mohr et al., 2001). Another study examining the relationship between divergent thinking and OT showed that scores on OT measured by Lovibond’s Object Sorting Test were positively correlated with originality of divergent thinking tasks, which were a reliable measure of creativity (Rawlings and Toogood, 1997). Similar to previous research, the present study employed categorization task to assess OT in terms of typicality rating and Reaction Time (RT) for the untypical exemplars. We developed the following two hypotheses on the relationships among OT, schizotypy, and creativity:

(1a) The high schizotypy group has significantly higher OT than the low schizotypy group, as indicated by higher rating and/or greater RT for untypical exemplars;
(1b) There is a positive correlation between OT and creativity measured by Alternative Uses Test and an extraterrestrials drawing task.

### Cognitive Inhibition, Schizotypy, and Creativity

Cognitive inhibitory control ability is a family of functions with three potentially separable processes—pre-potent response inhibition (PRI), resistance to distractor interference and resistance to proactive interference (Friedman and Miyake, 2004). Past studies have consistently reported a positive relationship between schizotypy and creativity but a negative relationship between schizotypy and cognitive inhibition (Beech et al., 1989; Moritz and Mass, 1997; Green and Williams, 1999; Kaplan and Lubow, 2011). Reduced cognitive inhibition was further hypothesized to link creativity to schizotypy (Eysenck, 1993; Acar and Sen, 2013; Fink et al., 2014b). However, the role of cognitive inhibition in the association between schizotypy and creativity has not yet been confirmed (Green and Williams, 1999; Crabtree and Green, 2016). This lack of the effect of cognitive inhibition on the creativity of schizotypical individuals may be because the positive association between the two variables has often been explained by reduced latent inhibition (LI), which refers to the phenomenon of neglecting target in test stage by rendering it as a distractor in the pre-exposure stage and represents the difficulty of processing a target that was previously irrelevant. It is reflected in the longer RT to pre-exposed target than to non-pre-exposed target. For example, 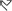 serves as a distractor in pre-exposure stage and transforms to a target in the test stage, then RT to 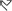 is slower than to novel target 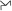 (Lubow et al., 2000).

However, some researchers have argued that the association between schizotypy and creativity may be more related to PRI indicated by Stroop interference effect because it is the active inhibition of pre-potent response (Friedman and Miyake, 2004), whereas LI, as the automatic resistance to proactive interference, does not give rise to awareness (Höfer et al., 1999). Stroop interference effect is indicated by slower RT to a task-incongruent stimulus (word: red; font color: green; task: name font color) than to a task-congruent stimulus (word: green; font color: green; task: name font color) (Edl et al., 2014). For this reason, PRI measured by Stroop interference effect was employed as the paradigm in the current study to conceptualize cognitive inhibition. Given schizotypy was related with reduced Stroop interference effect (Green and Williams, 1999) and Stroop interference effect was positively related with LI (Kaplan and Lubow, 2011), high level of cognitive inhibition is shown in reduced Stroop interference effect rather than lower level of LI in the current study.

Although some studies reported that inhibitory control hinders creative performance (Benedek et al., 2012a, 2014; Radel et al., 2015), other studies suggested that engaging in creative problem solving process requires the inhibition of past inappropriate ideas inducing fixation phenomena (Cassotti et al., 2016). Several studies have also revealed a positive association between reduced Stroop interference effect and creativity. For instance, reduced Stroop interference effect was positively correlated with enhanced verbal originality and fluency measured by the Torrance Test of Creative Thinking (Edl et al., 2014). The Stroop interference effect, which was considered a reversed indicator of inhibition, was also found to positively predict creative performance measured by divergent thinking tasks in a latent variable modeling study (Benedek et al., 2014). These findings imply that cognitive inhibition might suppress the interference of prominent ideas during the process of creating original ideas or products (Benedek et al., 2014). Based on the literature review, we made the following two hypotheses:

(2a) The high schizotypy group has higher cognitive inhibition than the lower schizotypy group, as indicated by reduced Stroop interference effect;
(2b) There is a positive correlation between cognitive inhibition measured by reduced Stroop interference effect and creativity.

Furthermore, according to the two sets of hypotheses mentioned earlier, we also hypothesized that OT and cognitive inhibition mediate the relationship between schizotypy and creativity.

## Materials and Methods

### Participants

Participants were selected from a subject pool consisting of 388 students from two colleges in Shanghai, China by using the Chinese version of Schizotypal Personality Questionnaire (SPQ) (Raine, 1991; Chen et al., 1997). Those who had personal history of mental disorders, neurological disorders, head trauma, and drug abuse and dependence were excluded from the study. Following several previous researchers (Meehl, 1990; Abraham and Windmann, 2008; Bedwell et al., 2011; Fink et al., 2014b; Chan et al., 2016; Koychev et al., 2016), we used categorical sampling approach and selected the high and low schizotypy participants based on the 10% base rate of schizotypy in the general population. More specifically, participants whose total scores on the SPQ fell into top tenth percentile were recruited as high schizotypy group and participants whose total scores on the SPQ fell into lowest tenth percentile were recruited as low schizotypy group (see **Table 1** for descriptive statistics of total SPQ and three SPQ factors in high schizotypy and low schizotypy groups). It may be more likely to detect the effect of schizotypy on creativity by using categorical sampling approach because “the maximum schizotypy effect would be achieved using a design that compares low and high schizotypes” (Koychev et al., 2016, p. 1). A global score of schizotypy was employed, following previous studies on schizophrenia that used the same practice (Abraham et al., 2007; Jones et al., 2011; Oertel-Knochel et al., 2013; Lui et al., 2016; Wang et al., 2017).

**Table 1:** Descriptive statistics of SPQ in the low and high schizotypy groups.

	SPQ total		SPQ Cognitive-Perceptual		SPQ Interpersonal		SPQ Disorganization	
	Mean (*SD*)	Minimum (Maximum)	Mean (*SD*)	Minimum (Maximum)	Mean (*SD*)	Minimum (Maximum)	Mean (*SD*)	Minimum (Maximum)
Low schizotypy	6.22 (2.42)	0 (9)	3.22 (1.88)	0 (8)	2.10 (1.73)	0 (7)	1.27 (1.16)	0 (6)
High schizotypy	44.95 (6.25)	38 (69)	20.35 (3.77)	10 (29)	18.46 (4.64)	8 (31)	10.38 (3.42)	5 (18)

In the final sample, there were 37 participants in the high schizotypy group, with 34 being female and 3 being male. The mean age of this group was 21.54 (*SD* = 1.33) and the mean education year was 14.14 (*SD* = 1.38). There were 41 participants in the low schizotypy group, with 35 being female and 6 being male. The mean age of this group was 21.73 (*SD* = 1.45) and the mean education year was 14.12 (*SD* = 1.36). There were no significant differences between two groups in terms of age [*t*_(76)_ = 0.61, *p* = 0.55], gender [*X^2^*_(1)_ = 0.26, *p* = 0.61], and years of education [*t*_(76)_ = 0.05, *p* = 0.96]. In addition, participants all reported normal color vision and normal or corrected-to-normal acuity before testing without taking drug or coffee. These participants formed a homogeneous sample because they had similar age, education background, and major, which minimizes the individual differences that are independent of schizotypy.

### Measures

#### Schizotypal Personality Questionnaire

The 74-item SPQ is one of the schizotypy self-report questionnaires that have been most extensively used in the field (Abraham and Windmann, 2008). This questionnaire was originally developed based on the criteria for schizotypal personality disorders that were specified in the Diagnosis and Statistical Manual of Mental Disorder (Revised 3rd Edition, DSM-III-R, Raine, 1991). It uses binary true/false format and assesses all the nine schizotypal traits that are contained in cognitive-perceptual (called as positive schizotypy), interpersonal (called as negative schizotypy), and disorganization factors. Some example items are “Do you ever suddenly feel distracted by distant sounds that you are not normally aware of?”, “Are your thoughts sometimes so strong that you can almost hear them?”. It was adapted by Chinese researchers (Chen et al., 1997) and has been used in Chinese context over the past two decades with good reliability and validity evidence. The reliability of the questionnaire in the current study was high (Cronbach’s α = 0.92).

#### Cognitive Inhibition

As mentioned earlier, PRI indicated by reduced Stroop Inference effect was used in the study as the paradigm of cognitive inhibition. The Stroop task was adapted from Bailey et al.’s (2010) study. Congruent [e.g., Chinese character for “red” (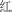) in red] and incongruent [e.g., Chinese character for “red”(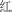) in green] color-words or strings of four Xs in colors red, blue, green or yellow were presented as stimuli at the center of the computer screen on a black background. The implementation of the Stroop task included three phases: key-mapping, practice, and test phase. The key-mapping phase included 40 trials with strings of four Xs as stimuli (10 trials for each color). During this phase, participants were instructed to verify the color of the stimulus as quickly and correctly as possible by pressing the key mapped to the color of the stimuli. The practice phase consisted of 12 congruent and 12 incongruent trials with color-words as stimuli. The test phase consisted of four blocks of 72 trials with color-words as stimuli. Each block included 36 congruent and 36 incongruent stimuli. The congruent and incongruent stimuli were pseudo-randomized in both practice and test phrases. When the participants gave press response, a trial was terminated. Incorrect response was given a feedback for 1000 millisecond (ms) and correct response was followed by a blank screen of 500 ms to ensure a high degree of Accuracy (ACC). The response, response time (RT), and ACC were recorded^3^. The test–retest reliability coefficients of the color-word Stroop were adequate: RT (0.86), interference effect (0.68).

#### Executive Functioning

Participants’ executive functioning (EF) was considered control variable in the study and included working memory capacity, shifting ability, and reasoning ability.

##### Working memory capacity

Working memory capacity was assessed by an operation span task that was adapted from Lin and Lien (2013). In this task, a set of equation-word pairs was presented on computer screen one by one. Participants were required to verify a simple math equation [e.g., (9/3) + 3 = 6] by pressing button (“1” for correct, “0” for wrong) while memorizing a two-character Chinese word [e.g., “
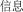
” (Information)]. Each equation-word pair remained on screen either until a verification response was given or for a maximum of 5 s. In order to ensure a high ACC, a feedback for 1 s was given after each response. At the end of each trial, participants were instructed to write down all the words presented in the whole trial on the computer. For instance, if there were four equation-word pairs in a trial, the participants would verify the equation and memorize the word from each pair concurrently and then write down the four words at the end of the trial. After practicing on three trials at a set size of 3 (i.e., three equation-word pairs), participants acted upon experimental trials at a set size from 2 to 7. There were three trials at each set size. If the participants could not accurately recall all the words for more than two trials at any given set size when correct equation verification was required, the test would stop. The highest set size accomplished by participants indicated the level of working memory capacity. The test–retest reliability for the operation span task was adequate (*r* = 0.77).

##### Shifting ability

Shifting ability in EF was assessed by number-letter task that was adapted from Miyake et al. (2000). A number-letter pair (e.g., 3E) was displayed in one of four quadrants on computer screen. The participants were required to judge whether the number in the pair was odd or even (3, 5, 6, and 9 for odd; 2, 4, 6, and 8 for even) when the pair was displayed in left or right upper quadrant. They were also required to judge whether the letter in the pair was a consonant or vowel (A, E, I, and U for vowel; G, K, M, and R for consonant) when the pair was displayed in left or right lower quadrant. The number-letter pair was displayed only in the two upper quadrants for the first block of 32 trials, only in the two lower quadrants for the second block of 32 trials, and in a clockwise rotation around all four quadrants for the third block of 128 trials. Ten practice trials were employed in the first and second blocks and 12 practice trials were used in the third block. Thus, the trials within the first and second blocks required no mental shifting, while half of the trials in the third block required the participants to shift between two types of operation (“number” or “letter”). The participants responded by pressing button “D” for “odd,” “F” for “even,” “J” for “vowel,” and “K” for “consonant.” To enhance ACC, there was a fixation for 500 ms before each response and a feedback for 800 ms after each response. The shifting ability was scored by the difference between the average RT of the trials in the third block that required a mental shift (trials from the upper left and lower right quadrants) and the average RT of the trials from the first two blocks that required no shift. The smaller the difference is, the better the shifting ability. The internal reliability estimate for the number-letter task was excellent (*r* = 0.91).

##### Reasoning ability

Reasoning ability was measured by Raven Advanced Progressive Matrics (RAPM), a non-verbal intelligence test. Only 18 out of 36 items were employed in the study, which were all the odd items in the test (Nusbaum and Silvia, 2011). All the items were presented in black ink on white background and ordered by the level of difficulty, with the easiest item being placed at first and the most difficult item being placed at last. The participants were required to identify the missing element to complete a pattern. They worked on 18 reasoning items for 12 min. The number of correctly answered items was the final score of each participant. The internal reliability estimate for RAPM was good (*r* = 0.87).

#### Overinclusive Thinking

Overinclusive thinking was assessed by a categorization task that was adapted from Chiu (2015). Typical and untypical exemplars of clothing category and vehicle category were used as stimuli in the task. Three typical and three untypical exemplars were included in each category. The clothing category included typical exemplars (i.e., suit, shirt, and pants) and untypical exemplars (i.e., ring, purse, and cane). The vehicle category also included typical exemplars (i.e., train, automobile, and bus) and untypical exemplars (i.e., camel, feet, and elevator). Each exemplar was presented on the computer screen consecutively until the participants gave a response. The participants were required to rate the typicality of exemplars on a 10-point Likert scale by pressing the corresponding number key, in which 0 indicated “definitely does not belong to the clothing (or vehicle) category” and 9 indicated “definitely belongs to the clothing (or vehicle) category.” The typicality rating and RT for each exemplar were recorded. The level of OT was indicated by the average score of typicality rating and RT for untypical exemplars of clothing and vehicle category (Chiu, 2015). Individuals with high OT were expected to have higher typicality rating or faster RT for untypical exemplars.

#### Creativity

Creativity was measured by multiple approaches because previous research showed that multiple ways of measuring creativity yield better results (Long, 2014a). First, participants’ verbal divergent thinking was assessed by Alternate Uses Test (AUT), a widely employed divergent thinking task (Beaty et al., 2014; Hao et al., 2014b). Although divergent thinking ability is not synonymous to creativity, divergent thinking tasks have been long employed to measure the originality and fluency of ideation (Runco, 1991; Long, 2014a; originality is preferred as an indicator of creativity because it has a conceptual relationship with standard definition of creativity, Forthmann et al., 2017). Participants were required to write down as many original uses as possible for four everyday objects (i.e., tire, barrel, pencil, and brick) within 3 min for each task. The creativity of the ideas generated from the AUT tasks were measured by originality and fluency (Guilford, 1967). Originality scores were assessed with subjective scoring method based on the Consensual Assessment Technique (CAT) (Amabile, 1982) because individuals usually have various interpretations for originality and how to score it is somewhat subjective (Long, 2014b). Each response was rated on a 5-point scale (1 = “Not original at all” 5 = “Highly original”) (Hao et al., 2014a) by six trained raters. The final originality score of each response was the mean of the six ratings. The originality of each task was the mean of the total originality scores of all the responses. The interrater reliability of the originality scores in the AUT tasks was satisfactory (Cronbach’s α = 0.70). Fluency scores were indicated by the total number of responses given by the participants for each AUT task. The originality and fluency scores of the four AUT tasks were averaged for every participant. The inter-rater reliability for AUT tasks were excellent (*r* = 0.88).

Participants’ creativity was also measured by extraterrestrials task that was developed by Ward (1994) aiming to tap the ability of breaking the boundaries of established concepts to create original products. Participants were given 20 min to draw imagined extraterrestrial creatures from front and side, respectively, and to briefly describe the drawings. They were told in the instruction that the extraterrestrial animals living on another planet were supposed to be different from the creatures on the Earth, so they can draw the creature as original as possible. Participants were also asked to report whether they had previous training on drawing and no significant difference was found between the two groups [*t*_(76)_ = 0.14, *p* = 0.89].

Participants’ drawings were assessed by two aspects: difference and originality. The difference score reflected the number of major differences between the extraterrestrial creatures drawn by participants and typical Earth creatures. The coding procedures of difference were in accordance with the approach of previous studies (Ward et al., 2004; Abraham and Windmann, 2008). Two coders assessed the difference by coding the presence or absence of five attributes in the drawings: bilateral symmetry, typical appendages (leg, arm, wing, and tail), typical sense organs (eye, mouth, nose, and ear), unusual appendages, and unusual sense organs. The presence or absence of any of the former three attributes was given a score of 0 or 1, and the presence or absence of either of the latter two attributes was given a score of 1 or 0. More specifically, the use of bilateral symmetry, one or more of the four typical appendages, and one or more of the four typical sense organs, was scored 0. For instance, an appendage was considered unusual and scored 1 if it contained atypical number (e.g., three legs), had extraordinary function (e.g., respiration with leg), or non-existent for Earth creatures (e.g., wheels). A sense organ was considered unusual and scored 1 if it contained atypical number (e.g., one eye), had fantastical function (e.g., sensing temperature up to 5 kilometers away), non-existent for Earth animals (e.g., built-in memory bank), or had odd arrangement of the sense organs (e.g., nose on the belly). The final difference score of the drawings ranged from 0 to 5. The final rating score was used when both coders were in agreement. When the two raters were not in agreement (less than 2% of all observations in the study), a third coder was consulted and the majority of the rating was used.

Originality score of the drawing was rated on a 7-point scale (Ward et al., 2004; Hao, 2010) (1 = “Not at all original” 7 = “Highly original”) by five trained raters based on the Consensual Assessment Technique (CAT) (Amabile, 1982). The originality scores provided by five raters were averaged for every participant. The interrater reliability of the originality scores was satisfactory (Cronbach’s *α* = 0.84).

### Procedure

This study was approved by the IRB of the university where the study was conducted. Written informed consents were obtained from participants prior to the study. A few weeks before the study, potential participants completed SPQ and the final sample of the participants were selected based on the results of SPQ. All the selected participants took part in the study in a group of 5–10 individuals in a quiet classroom. They were instructed to work on the Stroop task, Operation Span task, Number-letter task, Categorization task, Alternative uses test (AUT), Extraterrestrial animal task, and RAPM consecutively. This order was employed to minimize the effects of fatigue on RT and ACC in the first three tasks. The Stroop task, Operation Span task, Number-Letter task, and Categorization task were programmed in E-Prime software on computer. The response, RT and ACC were directly recorded in the computer. All of the other tasks were completed by using paper-and-pencil tests. The whole process lasted for 90 min. After the completion of the study, the participants were debriefed and rewarded 40 RMB (or about 6 dollars) as compensation. In order to be consistent, the protocol for the two groups was identical, and the administrations of the creativity assessment were performed by the same researchers.

### Data Analysis

All the statistical analyses were performed in IBM SPSS Version 24. Independent sample *t*-test and analyses of variance (ANOVA) were used to compare group differences in creativity indices, OT, and executive function tasks. Pearson correlation coefficients were used to examine the correlation between creativity indices and performance on OT and executive function tasks in the entire sample. Two mediation analyses were run for the effect of OT and cognitive inhibition on the relationship between schizotypy and creativity. The approaches to establish mediation suggested by Hayes (2009), including regression and bootstrapping, were used.

## Results

Means and standard deviations (SDs) of creativity tasks, OT task, cognitive inhibition, and other executive function tasks are presented in **Table 2**.

**Table 2:** Descriptive statistics of creativity, overinclusive thinking, cognitive inhibition, and executive functioning tasks in the low and high schizotypy groups.

Task	Low schizotypy *n* = 41 *M (SD)*	High schizotypy *n* = 37 *M (SD)*	*t_(76)_*	*p*
AUT originality	2.43 (0.26)	2.55 (0.26)	-2.01	0.048
AUT fluency	5.84 (2.67)	7.07 (3.04)	-1.90	0.062
Extraterrestrials difference	1.32 (0.97)	2.00 (1.08)	-2.92	0.005
Extraterrestrials originality	4.02 (1.15)	4.64 (1.05)	-2.46	0.016
Categorization RT: typical	2.31 s (1.01)	2.36 s (1.17)	-0.18	0.858
Categorization RT: untypical	3.58 s (1.81)	2.79 s (1.19)	2.26	0.027
Categorization Rating: typical	8.85 (0.35)	8.76 (0.40)	0.99	0.327
Categorization Rating: untypical	3.51 (1.83)	3.80 (1.90)	-0.67	0.503
Stroop RT: congruent	876.07 ms (220.25)	830.50 ms (237.91)	0.88	0.382
Stroop RT: incongruent	1034.6 ms (289.71)	934.01 ms (236.62)	1.67	0.099
Stroop interference effect	158.53 ms (92.17)	103.52 ms (61.64)	3.06	0.003
Stroop ACC	0.95 (0.04)	0.96 (0.02)	-2.31	0.024
Number-letter RT	491.15 ms (298.77)	419.51 ms (267.12)	1.14	0.257
Number-letter ACC	0.96 (0.04)	0.96 (0.04)	-0.34	0.732
Operation span	4.00 (0.78)	3.81 (0.94)	0.98	0.333
RAPM	10.78 (2.62)	11.30 (2.57)	-0.88	0.383

*AUT, Alternative Uses Test; RT, Reaction Time; ACC, accuracy; RAPM, Raven Advanced Progressive Matrix (intelligence); Categorization, Overinclusive Thinking; Number-letter, shifting ability; Operation span, working memory capacity.*

### Group Differences in Creativity Tasks

The results indicated that high schizotypy group scored significantly higher in originality of AUT [*t*_(76)_ = 2.01, *p* = 0.048, Cohen’s *d* = 0.46] and marginally significantly higher in fluency of AUT [*t*_(76)_ = 1.90, *p* = 0.062, Cohen’s *d* = 0.43] than the low schizotypy group. In addition, the high schizotypy group exhibited significantly higher scores on difference of extraterrestrials task [*t*_(76)_ = 2.92, *p* = 0.005, Cohen’s *d* = 0.66] and originality of extraterrestrials task [*t*_(76)_ = 2.46, *p* = 0.016, Cohen’s *d* = 0.56] than the low schizotypy group.

### Group Differences in OT Task

**FIGURE 1:**
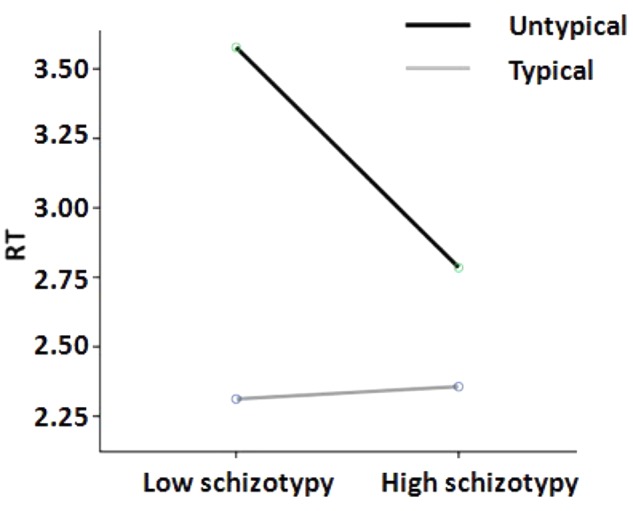
RT in seconds for untypical vs. typical exemplars in categorization task in low and high schizotypy groups.

Repeated measures ANOVA was employed to assess group differences in RT and rating of categorization task, with between subjects factor being group (low vs. high schizotypy group) and within subjects factor being typicality (typical vs. untypical exemplars). The results showed that the main effect of group on RT of the categorization task was non-significant [*F*_(1,76)_ = 2.37, *p* = 0.128, ${\text{η}}_{\text{p}}^{2}$ = 0.03]. However, there was a main effect of typicality on the RT of the categorization task [*F*_(1,77)_ = 21.60, *p <* 0.001, ${\text{η}}_{\text{p}}^{2}$ = 0.22], and participants responded faster to typical exemplars (*M* = 2.33 s, *SD* = 1.08 s) than to untypical exemplars (*M* = 3.20 s, *SD* = 1.59 s). More notably, there was a significant interaction of group and typicality on RT of categorization task [*F*_(1,76)_ = 5.28, *p* = 0.024, ${\text{η}}_{\text{p}}^{2}$ = 0.07]. The interaction indicated that the high schizotypy group responded faster for untypical exemplars (*M* = 2.79 s, *SD* = 1.19 s) than the low schizotypy group (*M* = 3.58 s, *SD* = 1.81 s), while there were no significant group differences for typical exemplars (see **Figure 1**).

In addition, there was a main effect of typicality on rating of categorization task [*F*_(1,153)_ = 571.38, *p <* 0.001, ${\text{η}}_{\text{p}}^{2}$ = 0.88], and participants rated higher for typical exemplars than for untypical exemplars. The main effect of group on rating of categorization task was not significant [*F*_(1,153)_ = 0.22, *p* = 0.643, ${\text{η}}_{\text{p}}^{2}$ = 0.003], and the interaction between group and typicality was not significant [*F*_(1,76)_ = 0.72, *p* = 0.398, ${\text{η}}_{\text{p}}^{2}$ = 0.01].

### Group Differences in Cognitive Inhibition and Other Executive Functioning Tasks

**FIGURE 2:**
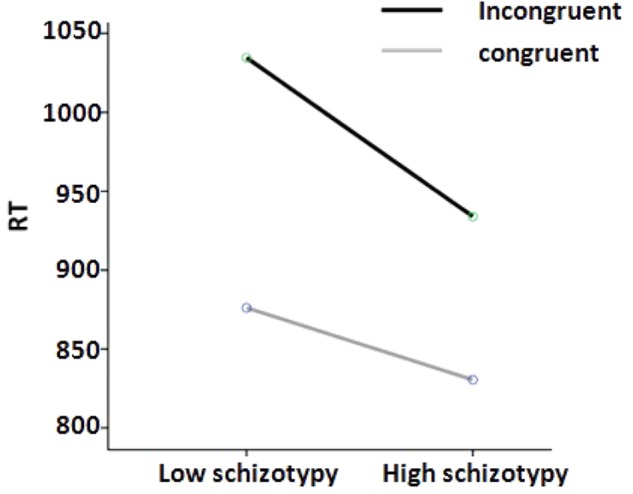
RT in milliseconds for congruent vs. incongruent trials in Stroop task in low and high schizotypy groups.

Concerning cognitive inhibition, repeated measures mixed effects ANOVA was employed to assess group differences in RT and ACC of Stroop interference task, with between subjects factor being group (low vs. high schizotypy group) and within subjects factor being congruence (congruent vs. incongruent trials). The results showed that there was no significant main effect of group on RT of Stroop task [*F*_(1,76)_ = 1.73, *p* = 0.192, ${\text{η}}_{\text{p}}^{2}$ = 0.02]. However, there was a main effect of congruence on RT of Stroop task [*F*_(1,77)_ = 196.72, *p <* 0.001, ${\text{η}}_{\text{p}}^{2}$ = 0.74]. This indicates a significant Stroop interference effect in that RT was slower for incongruent trials (*M* = 986.88 ms, *SD* = 268.95 ms) than incongruent trials (*M* = 854.45 ms, *SD* = 228.45 ms). More importantly, there was a significant group and congruence interaction on RT of Stroop task [*F*_(1,76)_ = 9.39, *p* = 0.003, ${\text{η}}_{\text{p}}^{2}$ = 0.11]. Specifically, the high schizotypy group responded faster for incongruent trials (*M* = 934.01 ms, *SD* = 236.62 ms) than the low schizotypy group (*M* = 1034.6 ms, *SD* = 289.71 ms), while there were no significant group differences in congruent trials (see **Figure 2**).

For ACC of Stroop interference task, significant main effect of congruence was observed [*F*_(1,77)_ = 150.47, *p <* 0.001, ${\text{η}}_{\text{p}}^{2}$= 0.66] in that there was lower ACC for incongruent trials (*M* = 0.94, *SD* = 0.04) than for congruent trials (*M* = 0.97, *SD* = 0.03). Moreover, there was a significant main effect of group on *ACC* of Stroop task [*F*_(1,76)_ = 5.40, *p* = 0.023, ${\text{η}}_{\text{p}}^{2}$ = 0.07] and there was higher *ACC* in high schizotypy group (*M* = 0.96, *SD* = 0.02) than in low schizotypy group (*M* = 0.95, *SD* = 0.04). However, there was no significant interaction between group and congruence on ACC of Stroop task [*F*_(1,76)_ = 0.65, *p* = 0.42, ${\text{η}}_{\text{p}}^{2}$ = 0.01]. In addition, there were no significant differences between low- and high-schizotypy groups in RT of number-letter task that measures shifting ability [*t*_(76)_ = 1.14, *p* = 0.257, Cohen’s *d* = 0.26], ACC of number-letter task [*t*_(76)_ = 0.34, *p* = 0.732, Cohen’s *d* = 0.08], working memory capacity [*t*_(76)_ = 0.98, *p* = 0.333, Cohen’s *d* = 0.21], and intelligence [*t*_(76)_ = 0.83, *p* = .407, Cohen’s *d* = 0.19].

### Mediation Effects of OT and Cognitive Inhibition Between Schizotypy and Creativity

According to the results of Pearson correlation coefficients among the variables (see **Table 3**), RT of categorization for untypical exemplars was negatively correlated with originality of AUT (*r* = -0.29, *p* = 0.010). The rating of categorization for untypical exemplars was positively correlated with fluency of AUT (*r* = 0.27, *p* = 0.016) and difference of extraterrestrials task (*r* = 0.27, *p* = 0.019). These results indicated that OT scores tended to be higher for more creative individuals. In contrast, the Stroop interference effect was negatively correlated with the originality of extraterrestrials task (*r* = -0.24, *p* = 0.037). This indicated that Stroop interference tended to be low and cognitive inhibition tended to be high for more creative individuals.

**Table 3:** Correlations between overinclusive thinking, cognitive inhibition, executive functioning, and creativity measures.

	1	2	3	4	5	6	7	8	9	10	11	12
(1) AUT Originality												
(2) AUT Fluency	0.03											
(3) Extrat Difference	-0.01	0.221										
(4) Extrat Originality	0.06	0.21	0.38^**^									
(5) Categor. RT : untypical	-0.29^*^	-0.07	0.02	-0.11								
(6) Categor. Rating : untypical	0.11	0.27^*^	0.27^*^	0.16	-0.07							
(7) Stroop inter. effect	-0.18	-0.13	-0.01	-0.24^*^	-0.00	0.04						
(8) Stroop ACC	-0.03	-0.12	-0.04	0.06	0.16	0.08	-0.21					
(9) Number-letter RT	0.13	-0.01	0.01	0.10	0.08	0.02	0.15	-0.14				
(10) Number-letter ACC	-0.05	-0.15	-0.06	0.10	0.23^*^	0.01	0.12	0.61^**^	0.11			
(11) Operation span	0.05	0.11	0.11	-0.14	0.07	0.11	0.04	0.16	0.27^*^	0.00		
(12) RAPM	0.21	0.01	0.17	0.14	0.05	0.08	-0.26^*^	0.08	-0.23^*^	0.00	0.27^*^	

*^∗^p < 0.05. ^∗∗^p < 0.01. Extrat., Extraterrials; Categor., Categorization; AUT, Alternative Uses Test; Extraterrials, creativity task; Categorization, Overinclusive Thinking task; Stroop, cognitive inhibition task; Number-letter, shifting ability task; Operation span, working memory capacity task; RAPM, intelligence task.*

**FIGURE 3:**
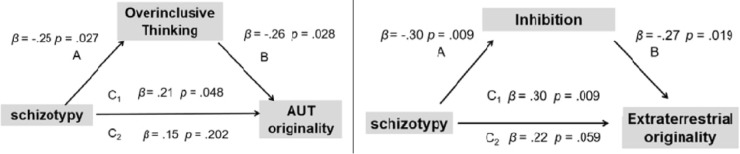
Mediation effects of overinclusive thinking, cognitive inhibition between schizotypy and creativity.

Three linear regressions and bootstrapping using Hayes macro PROCESS were further performed to establish mediation. In the PROCESS, a Bootstrap sample of 5,000 and Bias Corrected method were used to derive confidence interval for indirect effects. The regression results showed that the outcome variable, originality of AUT, was regressed on the predictor, schizotypy, and the path (Path C_1_) was significant (*β* = 0.21, *p* = 0.048) (see **Figure 3**). The relationship (Path A) between the predictor and mediator variable (i.e., OT) was significant (*β* = -0.25, *p* = 0.027), so was the relationship (Path B) between the mediator and outcome variables (*β* = -0.26, *p* = 0.028). Finally, when the outcome variable, originality of AUT, was regressed on both schizotypy and OT, the prediction effect of schizotypy (Path C_2_) became non-significant (*β* = 0.15, *p* = 0.202). Because the original regression coefficient reduced from 0.21 to 0.15, OT only partially mediated the relationship between schizotypy and originality of AUT. The standardized indirect effect of schizotypy on creativity via OT was significant, with the effect size being.03 [95% CI = 0.003,0.085].

The same analysis was used to assess the mediation effect of cognitive inhibition (i.e., Stroop interference effect) between schizotypy and creativity. Similar results were found, showing that cognitive inhibition partially mediated the relationship between schizotypy and originality of extraterrestrials task. However, the mediation role of the rating of categorization for untypical exemplars was not found because there was no significant difference between low and high schizotypy group in this variable. The standardized indirect effect of schizotypy on creativity via cognitive inhibition was significant, with the effect size being larger (0.18) than the effect of OT [95% CI = 0.029,0.471].

## Discussion

The current study examined the relationship among schizotypy, creativity, OT, and cognitive inhibition ability and found that high schizotypal individuals performed better on creativity tasks and had higher OT and cognitive inhibition. In addition, higher creativity was correlated with higher OT and cognitive inhibition. More notably, OT and cognitive inhibition partially mediated the relationship between schizotypy and creativity. These findings support our hypotheses on the relationships among these variables.

The study showed that high schizotypal individuals had higher OT as indicated by faster RT of typicality rating for untypical exemplars in categorization task. This suggests that high schizotypal individuals displayed advantages in automatic creative thinking process. This result is in line with previous findings. For example, Gianotti et al. (2001) found that individuals with high paranormal belief scores spent less association latency when generating words semantically related to given word pairs. Mohr et al. (2001) also reported that high schizotypy group measured by magical ideation were more likely to rate closer semantic relationship for unrelated or indirectly related word pairs than the low schizotypy group, which implies that high schizotypal individuals have the capacity of broadening conceptual boundary as reflected by higher OT.

The study also demonstrated that OT tended to be higher for individuals with more creative performance as indicated by originality and fluency of AUT as well as difference of extraterrestrials task. This positive association may be because OT, as a type of remote association ability, enabled individuals to produce a connection between remote semantic networks, thus, generating more original ideas (Mohr and Claridge, 2015). Moreover, individuals with better AUT performance showed faster judgment for the relatedness of the concepts and this speed advantage may contribute to the concept selection, which results in creative ideation (Vartanian et al., 2009).

The study further revealed that high schizotypal individuals tended to have higher cognitive inhibition as indicated by reduced Stroop interference effect. That is, higher cognitive inhibition was positively correlated with better creativity performance. This suggests that high schizotypal individuals are more likely to engage in the effortful/controlled creative thinking process. These results further replicate some of the previous studies. For instance, higher cognitive inhibition leads to higher creative performance in problem solving (Cassotti et al., 2016) and higher cognitive inhibition indicated by lower Stroop interference effect were positively correlated with better originality and fluency of creativity scores obtained from Torrance Tests of Creative Thinking (Edl et al., 2014). The reduced Stroop interference effect found in the study may be explained by the reduction in the effect of the regularities of the past experiences on the current perception, a mechanism called dissociation information process (Hemsley, 1993).

The results of this study further showed that other executive functions, including shifting ability, working memory capacity, and intelligence, were not significant predictors of creativity of high schizotypal individuals. However, they may serve as protective factors for these individuals so that they become people with creative potential rather than those with psychosis. This notion finds support from a previous review on shared vulnerability model of creativity and psychopathology, which suggested that highly creative individuals are protected by factors such as intelligence, working memory, and cognitive flexibility to make enlarged stimuli in conscious awareness that is manipulated and combined to generate unique ideas (Carson, 2011).

The most interesting findings of the current study were the partial mediation effects of OT and cognitive inhibition on the relationship between schizotypy and creativity. It is noted that better performance on AUT required the retrieval and combination of distantly related information (Fink et al., 2014b). Therefore, individuals with high OT might benefit from loose conceptual boundary and activate remote concepts when working on AUT. This mediation effect of OT on creativity also confirms the findings of a recent study, which indicated the increase of participants’ originality and fluency in the Torrance Creative Thinking Test after a training on OT (Chiu, 2015). Ward’s (1994) study also found that participants tend to generate imagined creatures with typical properties of animals on the Earth after the prompt of structured imagination. However, cognitive inhibition might supervise the originality of generated ideas and exclude common ideas (Beaty and Silvia, 2012; Edl et al., 2014). Thus, individuals with high cognitive inhibition could suppress the ordinary responses and retain the unique ones that are produced in creativity tasks. Therefore, high schizotypal individuals who have higher cognitive inhibition could override the existing category knowledge of the Earth creatures to produce more original extraterrestrials than did low schizotypal individuals (Ward, 1994).

The partial mediation effects of both OT and cognitive inhibition on the relationship between schizotypy and creativity further suggest that both cognitive processes, automatic/associative and effortful/controlled processes are involved in creativity performance of high schizotypal individuals. The results lend support for the dual process of creative thinking that highlights the important roles of these two processes in creative cognition (Allen and Thomas, 2011; Beaty et al., 2014, 2016). However, these two underlying processes may perform differently in specific stages and contexts of creative thinking (Allen and Thomas, 2011; Cheng et al., 2016). The automatic/associative process may drive response generation and problem searching, while effortful/controlled process may work on response evaluation and solution refining (Allen and Thomas, 2011; Beaty et al., 2016). Because AUT predominantly relies on idea generation (Radel et al., 2015), it might not be surprising to find that OT partially mediated the effect of schizotypy on creativity assessed by AUT. However, on the other hand, extraterrestrials task might require more response evaluation than response generation. Therefore, participants have to suppress the existing mental representation and category properties of Earth creatures when instructed to produce unique extraterrestrials that were different from Earth creatures (Ward, 1994). Moreover, individuals were more likely to employ automatic/associative process when completing tasks in a constrained length of time (Evans and Curtis-Holmes, 2005; Allen and Thomas, 2011). Because this study provided relatively longer time for extraterrestrials task (20 min) than for AUT (3 min), high schizotypal individuals might employ a more effortful/controlled process in extraterrestrials task than in AUT. This could avoid the “path of least resistance” (i.e., generate highly accessible ideas with least possible effort) and lead to a lower level of originality (Nijstad et al., 2010). However, this may also be related to the effect of speededness as recent studies have confirmed that less creative ideas were generated under the speeded condition and that the speededness is a significant predictor for mental speed shown in divergent thinking tasks (Preckel et al., 2011; Forthmann et al., in press).

There are a couple of limitations in the current study. First, the study only focused on two links that have been widely discussed in the field, OT and cognitive inhibition. Future research should investigate other potential links to these variables, such as, experience regression, unusual experiences (Acar and Sen, 2013), and neural hyper-connectivity (Carson, 2011). Second, the study employed a convenience sample and the generalizability of the results may be limited. In addition, the upper and lower 10% quantiles used to identify high- and low-schizotypy individuals for the sample might not reflect the base rate of a general population. Third, LI, as another mechanism to explain the link between schizotypy and creativity, was not examined simultaneously with Stroop interference effect. This is due to the following two considerations. First, this study aimed to explore the potential role of OT from the perspective of automatic/associative creative cognition and the role of cognitive inhibition from the perspective of effortful/controlled creative cognition. Second, too many tests may cause fatigue among participants and reduce accuracy in task completion.

### Implications for Rehabitation

Creative people tend to be described as people with mental illness (Andreasen, 1987; Macnaughton and Saunders, 2005; Glazer, 2009; Silvia and Kaufman, 2010; Kaufman, 2014). Schizophrenia, schizotypy personality disorder, and bipolar disorders are mental illnesses that are often thought to be closely related to creativity (Michalica and Hunt, 2013; Kaufman, 2014). Prior research has found the connection between creativity and these mental problems. It indicated that individuals who score high on schizotypy questionnaires tend to have unusual perceptions, odd ideas, inappropriate behaviors, and psychotic-like experiences. They are more likely to be described as eccentric than average people (Chapman et al., 1976; Eckblad and Chapman, 1983; Claridge, 1997; Fisher et al., 2004; Michalica and Hunt, 2013).

Individuals with schizophrenia or schizotypy show various degrees of deficits in many aspects, including cognitive, psychophysiological, neuro-psychological, personality, and morphological (Trestman et al., 1995; Park and McTigue, 1997). Because of these deficits, they show different symptoms, depending on the severity of the problems. For instance, they may experience hallucinations, delusions, magical thinking, social withdrawal, attentional difficulties, neuroticism, asocial behaviors, and impulsiveness (Claridge et al., 1996; Brod, 1997; Michalica and Hunt, 2013).

Over the past several decades, rehabitation programs have been developed to treat the impairment and symptoms of individuals with schizophrenia or schizotypy. For instance, Spaulding et al. (1994) employed a three-factor model to assess and treat cognitive and neuropsychological impairments in schizophrenia, including a vulnerability-linked first factor, episode-linked second factor, and psychosocial-amenable third factor. Sartory et al. (2003) implemented an adaptive, computerized training program with 42 patients having chronic schizophrenia for 45 sessions and found significant improvement in the attention, executive function, and verbal learning of the treatment group. Levaux et al. (2009) reported the success of the use of cognitive and ecological exercises in improving the schizophrenia patient’s sub-component of working memory.

However, the extensive impairment and symptoms of schizophrenia and schizotypal individuals pose challenges to the rehabitation, evidenced by several unsuccessful interventions (Pilling et al., 2002; Silverstein and Wilkniss, 2004). Most of the past interventions employed cognitive rehabitation approaches and mainly focused on cognitive impairments, which may negatively influence the effectiveness of the treatments. Silverstein and Wilkniss (2004) noted that schizophrenia rehabitation should systematically address other aspects, such as “motivation, self-esteem, and affective factors” (p. 679). The findings in the current and previous studies have consistently suggested that creativity functions as a protective factor that buffers individuals from negative influences. Although there are overlaps between creativity and schizophrenia and schizotypy, creative people are not always disorganized, asocial, or antisocial. In other words, there are ways that creative people use their unusual experiences constructively (Michalica and Hunt, 2013). It has also been indicated in the past research that creativity and creative thinking are important and effective coping strategies for individuals to become more resilient, particularly when faced with setbacks and difficulties (Carson et al., 1994; Davey et al., 2003; Edward and Warelow, 2005; Kitano and Lewis, 2005). Therefore, integrating creativity or creative activities in the future rehabitation programs for schizophrenia and schizotypy may effectively reduce the symptoms and disorders.

## Ethics Statement

This study was carried out in accordance with the recommendations of the guidelines of the Institutional Research Board at East China Normal University with written informed consent from all subjects. All subjects gave written informed consent in accordance with the Declaration of Helsinki. The protocol was approved by the Institutional Research Board at East China Normal University.

## Author Contributions

LW designed the study, collected and analyzed the data, and wrote the paper. HL interpreted the results and wrote and revised the paper. JP revised the paper. QW wrote and revised the paper. XX collected and analyzed the data. WP provided the idea of the study, interpreted the results, and revised the paper.

## Conflict of Interest Statement

The authors declare that the research was conducted in the absence of any commercial or financial relationships that could be construed as a potential conflict of interest.

## Footnotes

1 Schizotypy refers more specifically to positive schizotypy in the paper unless it is indicated otherwise.

2 Creativity in this paper mainly refers to everyday creativity (please see Kaufman and Beghetto, 2009 for the more details).

3 The false responses were not removed in this study. However, we analyzed the data removing the false responses based on the criterion of 3 standard deviations above/below the mean and the results remains the same as those not removing the false responses.
